# Impact of skin care on body image of aging people: A quasi-randomized pilot trial

**DOI:** 10.1016/j.heliyon.2023.e13230

**Published:** 2023-01-27

**Authors:** Masumi Nagae, Tsubasa Mitsutake, Maiko Sakamoto

**Affiliations:** a Department of Agro-Environmental Sciences, Faculty of Agriculture, Graduate School of Kyushu University, Fukuoka, Japan; b Department of Physical Therapy, Fukuoka International University of Health and Welfare, Fukuoka, Japan; c Division of Medical Education Development, Research & Education Center for Community Medicine, Faculty of Medicine, Saga University, Saga, Japan

**Keywords:** Body image, Well-being, Quality of life, Self-esteem, Skin care

## Abstract

**Aim:**

Support for various activities of daily living is essential for maintaining the health of residents in nursing homes. Although aging people who move to nursing homes often change their skin care habits, how these changes impact aging adults’ social and mental well-being remains unclear. This study aimed to evaluate the effects of facial skin care on aging residents’ self-body image, self-esteem, well-being, depressive symptoms and social cognitive function by a quasi-randomized controlled pilot trial in Japanese nursing homes.

**Method:**

Thirty-seven older adult women living in nursing homes took part in this quasi-randomized controlled pilot trial. Eighteen participants applied a skincare gel-cream to the face twice a day for three months, while 19 participants used no skincare products. Self-body image and psychological measures such as the Cutaneous Body Image Scale (CBIS), the Rosenberg Self-esteem Scale (RSES), Philadelphia Geriatric Center Morale Scale (PGCMS) and Geriatric Depression Scale (GDS) were used in each nursing home to evaluate the pre- and post-treatment scores. In addition, cognitive items of the Functional Independence Measure (FIM) were evaluated as social cognitive function at pre- and post-treatment.

**Results:**

There was a significant different change of the Cutaneous Body Image Scale scores (p = 0.045, r = 0.34) after three months between skin care group and control group. Although there were no clear significant differences in other psychological assessments, there was a higher number of them with positive changes in the skin care group compared to the control group.

**Conclusion:**

Skin care may help improve cutaneous self-body image and positive emotion in aging female residents of Japanese nursing homes.

## 1 Introduction

Aging is accompanied by physical, physiological, and psychological changes, which can reduce an individual’s capacity for daily activities, thereby decreasing quality of life (QOL). As life expectancy increases, maintaining satisfactory QOL for older adults, including the frail, is among the goals of care [1]. In Japan, the rate of aging in society is among the highest in the world; in addition, on average, Japanese men and women live for 22.14 and 15.98 years beyond the age of 65 years, respectively) [2]. In developing health actions for the elderly, we should consider the specificities of the aging process, which may influence both QOL and self-esteem [3]. Long-term care facilities are expected to maintain the QOL of older adults who have difficulty receiving family support. However, several activities of daily living (ADL) pertaining to skin care such as bathing and face cleansing for residents of Japanese nursing homes often differ from those of community-dwelling individuals [4,5]. Reasons for this includes that most ADL in nursing homes are easily affected by the level of human resources as well as individual independence of residents [6]. On the other hand, skin care in late adulthood is one of the cornerstones of nursing practice [7,8] and a growing concern among clinicians and caregivers [9,10]. However, long standing discussions about nursing home care quality have often focused on medical care rather than resident and family satisfaction [11], and most studies on skin care in nursing homes have concentrated on clinical care or outcomes of physical skin diseases [9,12]. Serious skin symptoms or infectious disease in nursing homes have also been reported in recent years [13]. In addition, more difficult factors such as clinical management with the COVID-19 pandemic have been added to these issues [14]. While medical care including skin care have received increasing attention, the mental health of older people in a society with restricted human contact has also been of interest [15]. Therefore, maintaining healthy skin is associated with better mental health and emotional well-being [16], even during the COVID-19 pandemic, and skin care should be an integral part of ADLs and multidimensional care among older adults.

Among skin care activities, facial skin care is often performed as a part of preparation before make-up from one’s younger years for many females. Aging residents who live in nursing homes often have difficulties using cosmetics but can perform facial skin care with skincare products. Facial skin care with skincare products of older residents in nursing homes may have an equivalent effect to make-up using cosmetics. Face perception promotes recognition by individuals, and provides information that facilitates social communication [17,18]. In addition, some studies report that the perception of one's face is an important part of body self-image that affects mental health [19]. Patzer [20] reported that people are perceived as more physically attractive after the use of facial cosmetics. In addition, Samson reported that visible skin condition affected perception of human facial appearance [21]. Kilpela et al. [22] explained that body image is a significant predictor of health and well-being in aging women. Barnett et al. reported that older adults lower in body image satisfaction may experience greater loneliness [23]. In the light of this previous research, the hypotheses in the present study were follows. First, practice of facial skin care with skincare products for older residents in nursing homes would have an equivalent effect to make-up using cosmetics, and would improve their self-image of the face. Second, by improving the self-image of the faces of residents in nursing homes, their communication and social cognitive function might improve, and health and happiness can also be expected to increase.

At present, few clinical trials on the impact of facial skin care in clinical practice have been conducted. Clinical decisions about nursing support in personal hygiene including skin care may also be poorly informed by evidence-based sources [24]. Clinical trials involving older people who live in nursing homes might contribute to the practice of nursing care which have been short of evidence for making clinical decisions. The purpose of this study was to evaluate the effect of facial skin care on older females’ self-image, self-esteem, well-being, depressive symptoms, and social cognitive function in Japanese nursing homes as a quasi-randomized pilot trial.

## 2 Methods

### 2.1 Intervention

This study, conducted from September 2017 to June 2018, was a quasi-randomized pilot trial of female elderly residents in nursing homes to compare psychological changes between two groups: a group which applied face skin care with a dedicated product, and a group which did not apply dedicated facial skin care product. The intervention was self-administered skin care routines applying a skincare gel-cream to the face twice a day for 3 months. The data were collected in Laboratory of Systematic Forest and Forest Products Sciences Department of Agro-Environmental Sciences, Faculty of Agriculture, Graduate School of Kyushu University.

### 2.2 Measures and outcomes

The intervention and survey questionnaires were completed in each private nursing home by one of six selected interviewers. These interviewers were previously trained by the lead researcher on questionnaire distribution and the ethical aspects of the research.

The Japanese version of the Cutaneous Body Image Scale (CBIS) [25] was used to evaluate self-image. The CBIS includes seven items, scored on a 10-point Likert scale, evaluating satisfaction with skin appearance, complexion, facial appearance, hair, and nails. Ratings of 0 indicate ‘not at all’ and rating 10 indicate ‘a great deal’ levels of satisfaction, respectively. The mean of seven rating items indicates the final score. Higher scores represent greater satisfaction while lower scores represent greater dissatisfaction. Cronbach’s alpha coefficient for the Japanese version of the CBIS was 0.88 for the non-clinical sample and 0.84 for the clinical sample [25].

The Rosenberg Self-esteem Scale (RSES) Japanese version [26], Philadelphia Geriatric Center Morale Scale (PGCMS) Japanese version [27] and Geriatric Depression Scale (GDS) [28] were used to evaluate the pre- and post-treatment scores to measure self-esteem, well-being and depressive symptoms respectively. Each evaluation was conducted in an interview style. In addition, cognitive Functional Independence Measure (FIM) [29] was used for pre- and post-treatment evaluation independent level of the cognitive function for each aging resident.

The RSES, which contains ten randomly distributed items, evaluated the self-esteem of the participants. Values from 1 to 5 points are assigned to each item, and the total score ranges from 10 to 50 points. In the Japanese version [27], high scores are associated with high self-esteem. Cronbach’s alpha coefficient for the Japanese version of RSES was reported 0.81 [30]. The PGCMS is designed to measure morale and is appropriate for very old or less competent individuals. The Japanese version [27] used was developed to evaluate well-being in clinical practice and research. Cronbach’s alpha coefficient for the Japanese version of PGCMS was reported 0.76 for 75–84 years old, 0.71 for 0.76, 0.71 for over 85 years old [31]. The GDS is among the most commonly used instruments for depression screening in later life; its Japanese version [28] is used in clinical practice, 5–9 points is judged to have depressive tendency, and 10 points or more is judged to be likely depression. Cronbach’s alpha coefficient for the Japanese version of PGCMS was 0.83 [28]. The FIM is an 18-item measurement tool that explores an individual's physical, psychological, and social function. It is used to assess the level of disability as well as the change in the patient’s status in response to rehabilitation or medical intervention [29]. Cronbach's alphas coefficient for the Japanese version was reported at 0.97 [32]. Since this study evaluated the effect of facial skin care on older females’ self-image and their associated psychology and social cognitive function, we adopted cognitive FIM. Cognitive FIM consists of communication and social cognition items.

The FIM was assessed by the care staff overseeing the residents who received additional training from the study ethicist. The group assignment of each participant was blinded to those care staff who assessed the FIM. The primary outcome was the change in the CBIS score, which evaluated cutaneous self-image of the participants pre- and post-treatment. The secondary outcomes were RSES, PGCMS, GDS scores and Cognitive FIM which evaluated self-esteem, well-being, depressive symptoms and social cognitive function associated with self-image respectively. The schedule of enrollment, intervention, and assessments are presented in Table 1.

**Table 1:**
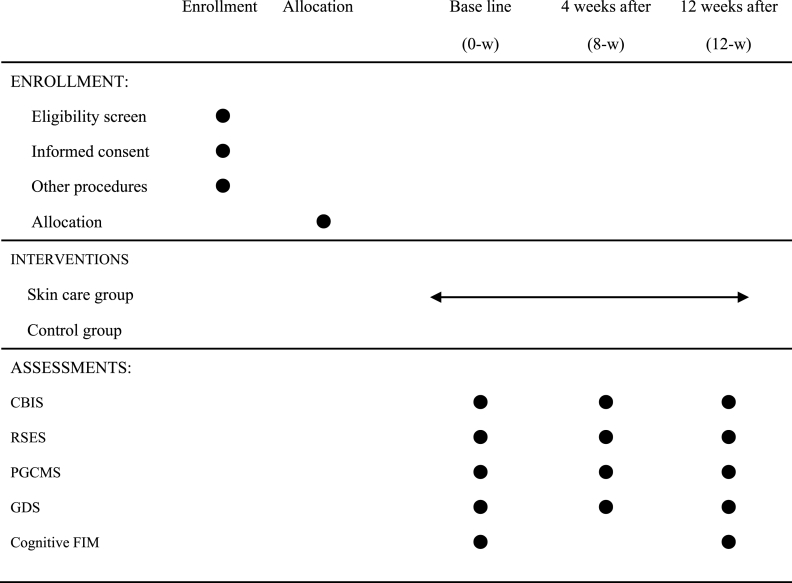
Schedule of enrollment, intervention, and assessments.

### 2.3 Participants and setting

From September 2017 to March 2018, we invited to 343 long-term nursing homes registered in a Japanese prefecture for self-skin care and mental health check programs by mail. Fourteen nursing homes volunteered to participate. Because there were no previous studies which measured CBIS that had reached statistical significance, we referred to a previous investigation [33] that had detected significant differences in RSES. The sample size was calculated with effect size (f) of 0.44, significance level (α) of 0.05, and statistical power (1−β) of 0.70, leading to total 36 subjects of two groups (18 subjects in each group). In addition, the number of participants was set at 42 (21 subjects in each group) to allow for dropouts and noncompliance with the protocol during the study period.

Residents, who provided written informed consent, were screened for eligibility for this study individually. The inclusion criteria included the ability of a participant to perform skin care rituals by herself and the lack of an established skin care routine or own skin care product. Residents with decreased lower limb function were eligible for this study, provided they could perform the skin care routine by themselves. All participants confirmed that they had no current skin care habits and were asked about their past skin care habits. Exclusion criteria included severe cognitive impairment, diagnosis of mental disorder, inability to engage in own skin care, and diagnosis of skin disease or sensitive skin. The participants assigned to the intervention group were asked to apply a skin care gel-cream to the face twice daily for 3 months. The participants assigned to the control group did not use any skin care products or perform any skin care beyond routine washing. Mental health assessments were performed pre-intervention, after four weeks, and post-intervention at the same time for both groups. Group assignment was randomized by a staff member who was independent from intervention staff.

Prior to the intervention, the measurement staff conducted a skin care and mental health seminar in each nursing home, and distributed a skin care gel-cream (Cola-rich Super All-in-One Beauty Gel Cream, Q'SAI Co., Ltd., Fukuoka, Japan) to each participant in the intervention group. This product was easy to use for older adults as it is a unique formulation which combines the functions of a lotion and a cream into a gel containing collagen, vitamin E, and other ingredients. Instructions for the quantity to apply to the face were given to each participant as per the product instructions and was confirmed every month by the assessment staff.

### 2.4 Randomization

Participants were designated for the group according to their age and the level of cognitive function at baseline. Cognitive function (level I, II, or higher) was measured using the Everyday-Life Independence Degree of Older Adults with Dementia, which is a Japanese government metric used for the certification of long-term care needs [34]. Levels I and II represented individuals who were independent, or independent but need to support from surroundings. Level Ⅲor higher represented with behavioral difficulties and need to care. Every enrollment and allocation of participants was performed while confirming the balance of composition of participants’ cognitive function level in both groups as a quasi-randomized trial.

### 2.5 Statistical analyses

SPSS (version 25.0, IBM Corp., Armonk. NY, USA) was used for data analysis. First, quantitative demographic variables were compared between the groups, using the independent *t*-test, Mann–Whitney *U* test, chi-square test, or Fisher’s exact test. Second, longitudinal data were compared using Friedman's test and multiple comparison test were performed to compare data of each assessment time within-subject factor. Last, Mann–Whitney *U* test was used for comparison between groups for difference values between pre- and post-intervention. Statistical significance was set at *p*-values of <0.05 and significant difference between groups were judged by *p*-values and effect size *r*.

## Ethical approval

The Faculty of Medicine, Saga University Sciences Committee on Ethics (29–26, 2017) and registered in the University Hospital Medical Information Network Clinical Trials Registry (UMIN-CTR ID: 000029316).

The study protocol complied with the ethical standards of the Declaration of Helsinki; it was approved by the Faculty of Medicine, Saga University Sciences Committee on Ethics (Approval number: 29–26, 2017) and registered in the University Hospital Medical Information Network Clinical Trials Registry (UMIN-CTR ID: 000029316). All participants were confirmed to have no diagnosis of skin disease or sensitive skin, and if a disorder such as an allergic reaction occurred, the use of product could be stopped immediately. In a case where it became necessary to see a doctor, the study representative was asked to contact and coordinate with the medical institution. All participants provided informed written consent before participating in the study.

### 2.6 Results

**Fig. 1:**
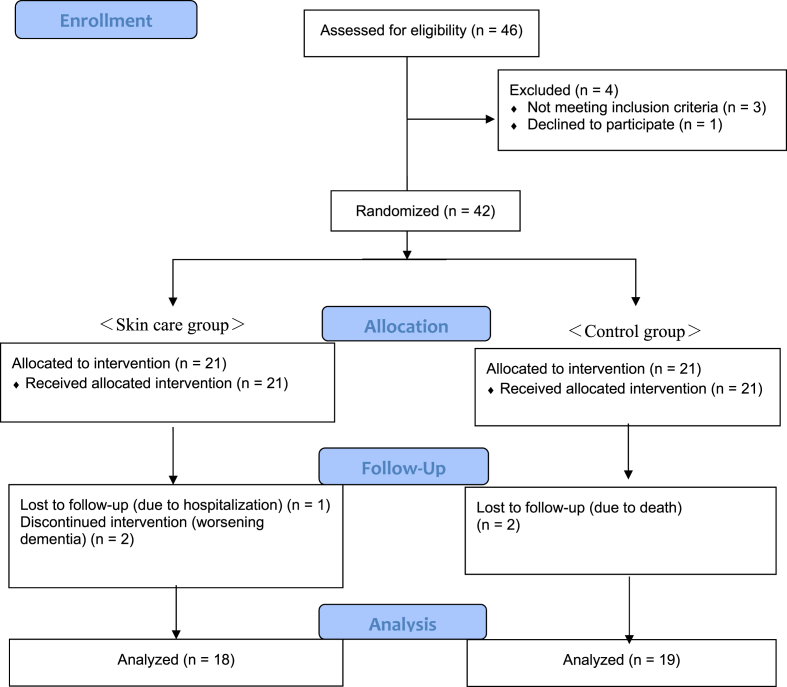
Flow diagram of the study participant selection.

A total of 42 older residents participated in this study for any of three months during the study period for total ten months from September 2017 to June 2018. Five participants dropped out during the study period. Three participants in the intervention group dropped out due to worsening dementia and hospitalization, while two participants of the control group passed away before the study was completed. Thirty-seven female residents completed the study, 18 in the skin care group and 19 in the control group (Fig. 1). During the three months of intervention, 18 residents in the Skin care group applied the gel-cream twice daily. Of these 18 residents, 12 performed skin care every time with care staff reminders, while 6 performed skin care independently.

The background characteristics of each group are presented in Table 2. The group assignment was based on the level of cognitive function and the mean age of participants at baseline. The mean age and standard deviation of the skin care group was 80.1 (SD 10.7) years, and that of the control group 86.1 (SD 7.3) years. There was no significant difference in age between groups. Including past skin care habits, there were no other significant differences in characteristics between the groups.

**Table 2:** Background characteristics of the intervention and control groups at baseline.

Items	Skin care (n = 18)		Control (n = 19)		P value*
	n (%)	Mean (SD) Median (0.25–0.75)	n (%)	Mean (SD) Median (0.25–0.75)	
**Age at baseline (years)**		80.9 (10.9)		86.1 (7.3)	0.098^a^
**Months in care home**		26.7 (14.9)		21.7 (13.5)	0.299^a^
**No. Of participants according to nursing home size**					
Small (<30 beds)	9 (50)		7 (37)		0.644^d^
Medium (30–45 beds)	4 (22)		7 (37)		
Large (≧45 beds)	5 (28)		5 (26)		
**Level of care**^**†**^		1.0 (1.0–2.3)		2.0 (1.0–3.0)	0.081^b^
**Cognitive function**^**‡**^		2.0 (1.0–3.0)		2.0 (1.0–3.0)	0.358^b^
**Health records**					
Bone fractures	5 (28)		9 (47)		0.219^c^
Internal diseases	5 (28)		4 (21)		0.714^d^
Cardiovascular disease	5 (28)		6 (32)		0.800^c^
Dementia/Higher brain dysfunction	2 (11)		4 (21)		0.660^d^
Depression/Anxiety	3 (17)		1 (5)		0.340^d^
**Frequency of skincare before living in nursing home**					
Daily	12 (67)		10 (53)		0.153^d^
Sometimes	2 (11)		0 (0)		
Rarely	4 (22)		9 (47)		
CBIS score at baseline		4.4 (1.6)		5.5 (1.6)	0.057^a^
RSES at baseline		35.5 (9.6)		33.2 (8.2)	0.439^a^
PGCMS at baseline		10.4 (2.3)		10.0 (4.2)	0.716^a^
GDS at baseline		9.9 (2.5)		8.7 (3.2)	0.224^a^
Cognitive FIM at baseline		29.5 (25.8–32.3)		31.0 (25.0–34.0)	0.620^b^

*P values were determined using the ^a^Independent *t*-test, ^b^Mann–Whitney *U* test, ^c^chi-square test, ^d^ Fisher's exact test for comparisons between the Skin care and control group.

SD: standard deviation; CBIS, Japanese version of the Cutaneous Body Image Scale; FIM, Functional Independence Measure; GDS, Geriatric Depression Scale; PGCMS, Philadelphia Geriatric Center Morale Scale; Geriatric Centre Morale Scale; RSES, The Rosenberg Self-esteem Scale.

† Level of care was evaluated based on the primary nursing care requirement authorization, which is a 7-level graded system under the health insurance scheme of the Ministry of Health, Labor, and Welfare of Japan.

‡ Level of cognitive function was evaluated based on the independence degree of daily living among older adults with dementia, which is a 9-level graded system, under the health insurance scheme of the Ministry of Health, Labor, and Welfare of Japan.

### 2.7 Effects of skin care on self-image and well-being

In the present study, since the sample size was not large enough to adopt parametric tests, non-parametric tests were performed. Longitudinal data of three assessment times of effects of skin care on self-image and other mental and cognitive functions were compared within each group.

**Fig. 2:**
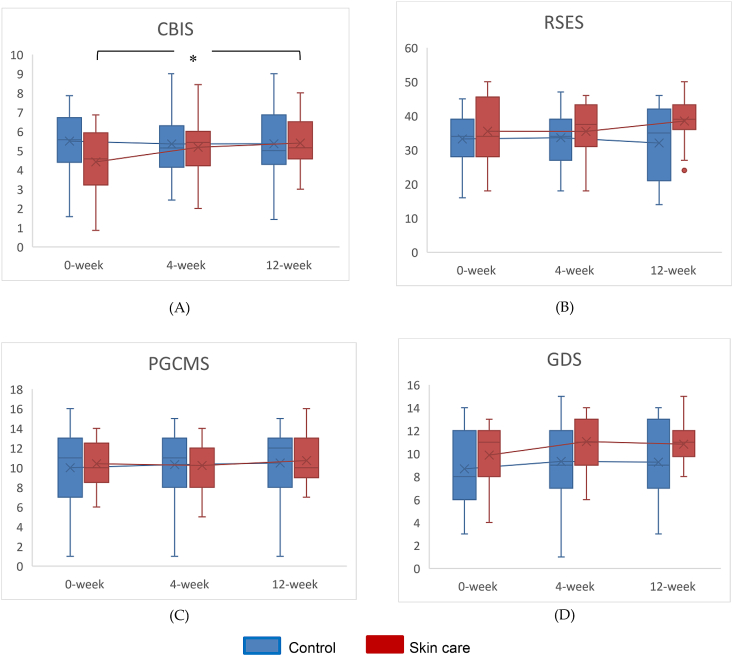
Longitudinal changes of data of each measure scale. (A) The Cutaneous Body Image Scale (CBIS), (B) The Rosenberg Self-esteem Scale (RSES), (C) Philadelphia Geriatric Center Morale Scale (PGCMS), (D) Geriatric Depression Scale (GDS). Dots beyond the bounds of the whiskers denote outliers. Cross marks, horizontal line indicate means, medians, for each group. Asterisks indicate significant difference of intragroup after the intervention, *: P < 0.05 (0-week vs 12-week).

First, for self-image measured using the CBIS, the skin care group showed a significant change in CBIS score (p = 0.026), while the control group score had no significant change (p = 0.881). After multiple comparison test were performed, there was a significant difference between the data at 0 week and those at 12th week within the skin care group (p = 0.031) (Fig. 2-A). In addition, there was a significant difference in changes of CBIS score between two groups over the 3-month period (*p* = 0.045, effect size *r* = −0.33) (Table 3). Next, RSES showed no significant changed in both groups, (p = 0.230 for the Skincare group, p = 0.961 for the control group) (Fig. 2-B). There was no significant difference in changes of RSES score between two groups over the 3-month period, but an effect of a small size for difference between groups was shown (p = 0.107, effect size r = 0.27) (Table 3). Similarly, PGCMS scales showed no significant change in either group, (p = 0.241 for the Skincare group, p = 0.423 for the control group) (Fig. 2-C) and there was no significant difference in changes of PGCMS score between two groups over the 3-month period (p = 0.827, effect size r = −0.04). (Table 3). Equally, GDS scores showed no significant change in either group, (p = 0.278 for the Skincare group, p = 0.285 for the control group) (Fig. 2-D) and there was no significant difference in changes of GDS score between two groups over the 3-month period (p = 0.594, effect size effect size r = 0.09) (Table 3). Finally, Cognitive FIM scores showed no significant difference in changes between two groups over the 3-month period but an effect of small level for difference between groups was shown (p = 0.118, effect size r = 0.27) (Table 3).

**Table 3:** Comparison of changed value of 12 weeks between control group with skin care group.

Items	Intervention	Control	p value	Effect size r
PGCMS	1.00 (−1.50–3.00)	1.00 (−1.00–1.00)	0.827	−0.04
RSES	4.00 (−3.00–7.00)	−3.00 (−6.00–6.00)	0.107	0.27
GDS	1.00 (−0.50–2.00)	1.00 (−1.00–2.00)	0.594	0.09
CBIS	1.14 (0.29–2.00)	−0.43 (−1.36–1.07)	0.045	0.34
Cognitive FIM	0.00 (0.00–1.00)	0.00 (−2.00–0.00)	0.118	0.27

The data are presented as the median (0.25–0.75), P values were determined using Mann–Whitney *U* test for comparisons between the Skin care and control group.

Under the conditions of this study, no side effects or adverse events were observed throughout the study period.

## 3 Discussion

In the present study, the effects of skin care on Japanese nursing home female residents’ self-image, self-esteem, well-being, depressive symptoms and cognitive function were evaluated. The practice of self skin care using the gel-cream twice daily for 3 months was associated with having increased the cutaneous self-image of aging residents in the skin care group.

Samson et al. reported that visible skin condition influences perception and attractiveness judgements [21]. Although we did not evaluate improving cutaneous quality quantitatively, there was a significant difference in changes between the two groups for cutaneous body image after the intervention. The subjective evaluation of patients’ skin condition might have resulted from improvements to the skin observed after three months. Consequently, the cutaneous self-image of the skin care group may have increased more than the controlled group. Regarding RESS which evaluated self-esteem, there was no significant difference between two groups over the 3-month period, but an effect of small size was shown for it. The skin care intervention may have been associated with their self-image and may have been associated with a positive effect on self-esteem. A previous study reported that older people in nursing homes had higher depression and higher anxiety, and the levels of self-esteem associated with levels of depression [35]. However, this study did not show a strong association between levels of self-esteem and levels of PGCMS or those of GDS. This may be due to two facts that most of the participants were older people dependent on ADL and the sample size was small. Conradsson et al. reported that depressive symptoms or psychological well-being among older people dependent on ADL and living in residential care facilities was not influenced by a functional exercise intervention [36]. Although the intervention in this study was autonomous self-care routines rather than exercise, most of participants were dependent on ADL, did not improve their physical ADL significantly, and therefore might have not changed their depressive symptoms or psychological well-being easily. A previous study indicated positive results that an art therapy intervention with 50 Korean American older adults who were independent promoted healthy aging by reducing negative emotions, improving self-esteem (RSES), and decreasing anxiety [37]. Conradsson et al. reported that significant improvement in PGCMS score in a total of 100 dementia older people living in residential care facilities by a high-intensity functional exercise program [36]. Liao et al. reported that an intervention which combined music and Tai Chi improved GDS score significantly among 55 community-dwelling older persons [38]. By contrast, most of the participants in this study were dependent on ADL. Moreover, since sample size and target analyses were lower than those of previous studies, we cannot deny that insignificant results might have been affected by those factors.

The Cognitive FIM evaluated the level of cognitive ADL, and simultaneously evaluated communication and social cognitive scores. Although the difference of change for three months between two groups did not reach significance, the cognitive function scores of the Skin care group were maintained, while those of the control group decreased slightly. In addition, an effect of small level was shown for the difference between two groups over the 3-month period. In this study, although the intervention period was only three months, the slightly different direction of change in Cognitive FIM between two groups was shown. The effect of skin care routine on cognitive function might merit further and more detailed investigations.

In this study, facial skin care changed the facial cutaneous self-image, which may be important for the elderly. A previous study reported that aging women who were more satisfied with the cosmetic features of their bodies were more socially engaged [18]. Another previous study suggested that body image is a significant predictor of health and well-being in aging women [17]. On the other hand, it was reported that women may change their body-related concerns with age, wherein their value of body shifts to different aspects, such as functional aspects, rather than cosmetic or appearance-related features [39]. Although there are opposite discussions of importance or self-concern with facial cutaneous image in aging women, the present study showed that changes to the CBIS scores may affect their self-esteem. Even if appearance becomes less important to women over time, different tendency of changes in RSES score between groups after 3 months was found in the present study.

In previous studies, satisfaction with life and self-esteem were not associated with frailty [40]. Positive self-esteem may be a protective factor of social behavior in frail subjects who need care and support [41]. The skin care group maintained their cognitive FIM throughout the study period. Unlike in the case of community-dwelling older people, residents of nursing homes dependent on ADL may have difficulty in accessing opportunities to increase social function. However, the positive self-esteem of skin care group may have promoted their expression or interaction in their residing nursing homes, and therefore social cognitive function might have been maintained for this group. Regarding QOL in the nursing home, dignity remained a significant predictor of older residents’ satisfaction [39]. Lee et al. reported about humanistic care indicators including holistic skin care based on the individual needs of residents of nursing homes [40]. Skin care should be carried out according to the level of support required for older people. Even older people with decreased lower limb function who use a wheelchair can still perform self-care autonomously. If one’s cognitive function is declining, one may be able to perform skin care with care staff reminders.

### 3.1 Limitations

This study had some limitations. First, since the number of participants in this study was low, after some dropouts occurred, the analysis target number was further decreased. The sample size was small, and each participant’s institution could not be considered or compared as a block. This study resulted in difficulty in making direct comparisons with those of previous studies’ [[36], [37], [38]] Magid, Galenbeck and Levy [42] stated that requiring nursing home residents to give consent to participate may result in recruitment of a sample that is not fully representative of the population who would receive the intervention in usual care, especially given the prevalence of dementia in nursing homes. In this study, all participants were required to provide written informed consent, and one of the inclusion criteria was having the ability to perform skin care rituals. The number of older eligible residents who could participate during the period of study was limited, and made it difficult to show causality clearly. Although group assignment was blinded to intervention staff, it was difficult to blind perfectly to participants and researchers because of the length of the intervention (3 months) and because of study characteristics. Second, assessment of the outcomes was based on subjective evaluations by the participants and their caregivers. There is a possibility that individual differences of evaluation and mental and physical condition at each assessment time influenced the evaluation. Third, the gel-cream is commonly used by older women in Japan and is not available in other countries; hence, the results may not be generalizable elsewhere. Although the characteristics of the product might have played an important role in the results, they were not compared to those of other products. In addition, the psychological effects of the changes in the cutaneous body image among the study participants may have included confidence and satisfaction from their continuation of skin care. Furthermore, we evaluated the psychological effect of skin care practice, but there was no such evaluation of dermatologic effects. Future studies should examine the impact of changes to the cutaneous body image and dermatological development on the psychological health of older adults. Lastly, all participants were women who lived in nursing homes in Japan; Future studies should include all sexes, different countries, regions, and cultural backgrounds as well as different skin care products.

## 4 Conclusion

Nursing home residents may lack access to experiences and opportunities for participation, self-esteem and well-being in frail older adults making it difficult to compare with those in younger generations. This study suggested the association that female older residents of Japanese nursing homes living in limited social environment might improve cutaneous self-body image and develop their self-esteem by their own self skin care. As mentioned in the limitations, there remains a suspicion that the data in this study were not representative of all applicable older residents including those with decreased cognitive function. However, to the best of our knowledge, this study was the first study evaluated the effects of facial skin care on aging residents by a quasi-randomized controlled trial in Japanese nursing homes. Although the generalizability of the findings may be limited in the present study, the results obtained in clinical trials are considered to be important data and necessary pilot studies to create a standard for judgment in clinical practice.

In recent years, several countries have focused on maximizing QOL of elder residents of nursing homes. Daily care in nursing practice of older people should be focused on not only clinical care regarding diseases but also better individual mental and emotional well-being. As one of the means to live with dignity in oneself throughout life, it is necessary to recognize the importance of personal cosmetic care in clinical practice. While more difficult clinical management with the COVID-19 pandemic has become necessary, skin care should also be kept in focus for increasing the dignity of older people in the society restricted with human contact.

## Author contribution statement

Masumi Nagae: Conceived and designed the experiments; Performed the experiments; Analyzed and interpreted the data; Contributed reagents, materials, analysis tools or data; Wrote the paper.

Maiko Sakamoto: Conceived and designed the experiments; Wrote the paper.

Tsubasa Mitsutake: Conceived and designed the experiments.

## Funding statement

This work was supported by 10.13039/501100001691JSPS KAKENHI, Japan, Grant Number JP17K09218.

## Data availability statement

The authors do not have permission to share data.

## Declaration of interest’s statement

The authors declare no conflict of interest.
